# Short-Term Efficacy of Pudilan Keyanning Toothpaste in Treatment of Minor Recurrent Aphthous Ulcers

**DOI:** 10.1155/2016/9125327

**Published:** 2016-11-14

**Authors:** Yingming Yang, Tao Zhang, Zibo Dong, Yang Wu, Xiao Hong, Tao Hu

**Affiliations:** ^1^Department of Preventive Dentistry, West China School and Hospital of Stomatology, Sichuan University, Chengdu 610041, China; ^2^Nanjing University of Chinese Medicine, Nanjing 210023, China

## Abstract

*Aim*. To examine the potential of Pudilan Keyanning toothpaste (PKT) to treat minor aphthous ulcers (MiAU).* Method*. A double-blind clinical trial was conducted in which 80 volunteers were randomly assigned to the PKT group (*N* = 40) or the control group (*N* = 40). The control group used a placebo toothpaste containing no Pudilan extract. At baseline, after 3 days, and after 6 days the following parameters were recorded for the target ulcers: healing rate, healing period, pain (visual analogue scale, VAS), areas of the target ulcerated lesions, degree of exudation, and hyperemia.* Results*. At the end of the study, the healing rate in the PKT group was 80%, compared to 50% in the control group (*p* < 0.05). At day 6, the VAS scores, ulcer area, degree of exudation, and hyperemia were significantly different between the two groups, with better performance observed in the PKT group (*p* < 0.05).* Conclusion*. PKT toothpaste appears to promote effective healing of MiAU.

## 1. Introduction

Recurrent aphthous ulcers (RAUs), also known as recurrent oral ulcers (ROUs), are a common oral mucosal disorder, with a reported prevalence of 25% to 80% [1–5]. It usually occurs in the nonkeratinized oral mucosa, with clinical manifestations including mucosal depression in the affected area, which is often covered by a yellowish-grey pseudomembrane, a burning sensation, and hyperemia or slight edema of the adjacent mucosa [6]. RAUs are characterized by periodic recurrence and tend to be self-limiting. Patients suffering from RAUs usually have difficulty chewing, drinking, swallowing, and speaking. In severe cases, RAUs disturb a patients' sleep and can even induce some systemic reactions, such as fever [7]. RAUs can be classified into three types: minor aphthous ulcers (MiAUs), major aphthous ulcers (MAUs), and herpetiform ulcers (HUs) [8]. Of these, MiAUs usually manifest as lesions with a diameter smaller than 1 cm and last for 4–14 days [9].

Treatment for RAU mainly includes localized or general medications [10]. Glucocorticoids, which have proven efficacy and safety, are often among the first choices for localized treatment. However, their long-term use may facilitate oral infection with* Candida albicans*. Other localized treatments include painkillers, antiseptic drugs, and prohealing drugs. Systemic medications such as prednisone, colchicine, and azathioprine are usually given to patients with severe or persistent symptoms [11–14]. A variety of formulations have been used including tablets, patches, mouth rinses, liquids, pastes, and gels [15, 16].

MiAUs make up 80–90% of all RAU cases [17, 18] and the most common symptom of MiAUs is pain that becomes worse when a patient tries to eat, drink, or speak, which consequently affects their quality of life. Hence, the focus for treatment lies in providing analgesia and promoting lesion healing. Several reports suggest that glucocorticoids and chlorhexidine mouth rinses can alleviate pain during a MiAU episode [19, 20]. Other new treatment methods, including ultrasonic and laser treatment, also exist [21, 22]; however, the efficacy and the side-effects of these treatment strategies require further investigation. As the etiology of RAU remains unclear, there is debate about the best treatment method [23].

Although reports on the efficacy of RAU treatment with herbal medicines are relatively rare in the literature [24], it is believed that the complex synergistic effect between different individual components in an herbal formulation can enhance the overall treatment efficacy [25, 26].

Pudilan Keyanning toothpaste (PKT) contains Pudilan extract, which is a combination of extracts of dandelion, isatis root, Bunge corydalis herb,* Scutellaria baicalensis*, gallnut, and propolis and all the individual components are traditional Chinese medicines that have been used for hundreds of years. One study showed satisfactory short-term treatment effects in MiAU patients with Pudilan oral liquid [27]. However, the addition of Pudilan extract to toothpaste to treat RAU has not been previously reported. The aim of the present study was to examine the potential of PKT to treat MiAUs.

## 2. Materials and Methods

The study protocol was designed according to the principles of the Declaration of Helsinki and related regulations for clinical studies in China and was approved by the Ethics Committee of West China Hospital of Stomatology, Sichuan University (WCHSIRB-D-2015-081-R1). This trial was registered as a clinical study (Registration No. ChiCTR-IOR-16007969: efficacy of a new dentifrice containing Chinese herb extracts for recurrent aphthous ulcer: a double-blind randomized placebo parallel controlled trial). All participants in the study were volunteers.

### 2.1. Study Design

The study was a randomized, double-blind, placebo-controlled clinical trial conducted as a superiority test.

Altogether, there were 80 participants who, through random codes produced by SAS software, were block-randomized into the PKT group or the control group. Each group comprised 40 people.

#### 2.1.1. Inclusion Criteria

To be included in the trial volunteers must

- have expressed their agreement to participate by signing the informed consent form;
- be 18 to 62 years old;
- have been confirmed as having MiAU with 1–3 ulcerous lesions and a maximum lesion diameter < 10 mm;
- have had MiAUs diagnosed at least 6 months prior to this study and not have required medication for more than 5 days to recover from any episode;
- have had recent ulcerous lesions occurring within 48 h prior to the study and not received any treatment;
- have had lesions located conveniently for clinical examination and judgment.

#### 2.1.2. Exclusion Criteria

- Volunteers that had hepatic, renal, or other severe systemic diseases
- Volunteers that had an abnormal index value greater than 30% or of clinical significance
- Atopy or history of allergic reactions to the study product components
- Using glucocorticoids or other immunomodulatory drugs within the previous month
- Having used other drugs within the 48 h prior to the study that might interfere with the healing of RAU
- Women who were already pregnant, or planning to become pregnant, or breast feeding at the time of the study, or using steroid contraceptives
- Participation in other similar clinical trials within the previous 3 months
- Having had simultaneous MAU, HU, Behcet's disease, premenstrual ulcers, or other serious oral mucosal diseases
- Wearing orthodontic appliances or retainers in direct contact with the ulcer surface
- Having psychiatric disorders
- Smoking or being alcoholic [28]

#### 2.1.3. Treatment Method

Toothpaste (120 g/tube) used in the both study groups was produced by JumpCan Group Co. Ltd. (Baotaiwan, West Daqing Road, Taixing 225441, China). For the PKT group, the toothpaste ingredients were based on an authorized China Invention Patent (No. ZL201410199666.4) and the toothpaste contained Pudilan extract (concentration range 0.8–2.2%), Baicalin extract, polyethylene glycol, glycerol, sorbitol, xanthan gum, sodium carboxymethylcellulose, glucide, sodium lauryl sulfate, silica gel, silica gel type 153, silica gel type 203, flavours, soluble propylhydroxybenzoate, nipabutyl sodium, and sodium hydrate. The placebo toothpaste contained similar ingredients, but without Pudilan extract, and was adjusted for color and flavor with chemicals with no clinical effects.

The PKT and placebo toothpastes were given random codes and each participant received a random tube. The participants were asked to brush their teeth for 2-3 minutes, twice a day (in the morning and evening), each time covering two thirds the length of the toothbrush provided. After brushing the participants were asked to apply a little toothpaste to cover the ulcer surface with a cotton swab provided.

#### 2.1.4. Parameters Observed

If there were multiple ulcerous lesions, only the largest was included as the target ulcer. At baseline, 3 days later and 6 days later, the following parameters were recorded.

*Main Index*. The healing rate of the target ulcers was recorded 6 days after the baseline.

*Minor Indices*

- Healing period: time from the onset of the ulcer to its healing
- Pain: pain was measured using visual analogue scale (VAS) scores that ranged from 0 (not painful at all) to 10 (the maximum level of pain) [29, 30] All participants were required to record their VAS scores daily. VAS scores at the baseline, 3 days later, and 6 days later were used for statistical analyses.
- Area of the target ulcer
- Degree of exudation: 0, no exudate; 1, mild surface moisture; 2, mild yellowish-grey exudate; 3, presence of significant exudate and pseudomembrane formation
- Degree of hyperemia: 0, no hyperemia; 1, reddish; 2, deep red; 3, purplish red [31, 32]

### 2.2. Statistical Analyses

Statistical analyses were performed with SAS 9.1 software. Various baseline data were compared between the PKT and the control groups, as follows.

(i) Age, height, and weight were compared using the *t* test.
(ii) Sex and nationality were compared using the chi-squared test and Fisher's exact test.

### 2.3. Treatment Efficacy

Treatment efficacy was analyzed as follows.

(i) The healing rate of the target ulcers in the two groups was compared using the chi-squared test.
(ii) Healing period: the median healing times in the two groups were compared by the log-rank test.
(iii) The Wilcoxon rank test was used to compare VAS scores.
(iv) The area of the target ulcer was compared by *t* test and Wilcoxon rank test.
(v) Exudation and hyperemia of the target ulcer were compared with the Wilcoxon rank test, the chi-squared test, and Fisher's exact test.

#### 2.3.1. Analysis of Confounding Factors

*Simultaneous Medication*. All participants were required to list all drugs they had used during the study period. Chi-squared tests and Fisher's exact test were used to compare the two study groups.

*Participant Compliance*. Participant compliance was determined by the ratio of the actual number of times teeth were brushed to the number of planned brushings. The Wilcoxon rank test was used to compare differences in compliance.

## 3. Results

### 3.1. Comparability Analysis

Demographic baseline data (including age, sex, and nationality) and target ulcer status at baseline (area, VAS scores, exudation, and hyperemia) were comparable between the two test groups (*t* test, chi-squared test, and Fisher's exact test, *p* > 0.05).

### 3.2. Treatment Efficacy Analysis

#### 3.2.1. Healing Rate

At 6 days after beginning of the treatment, the healing rate in the PKT group was 80%, which was significantly higher than the 50% in the control group (chi-squared test, *p* = 0.049). The superiority test revealed similar findings; see Table 1.

#### 3.2.2. Healing Period

The median healing times of the two groups were compared by the log-rank test and were not significantly different (*p* > 0.05).

#### 3.2.3. VAS Scores

**Figure 1:**
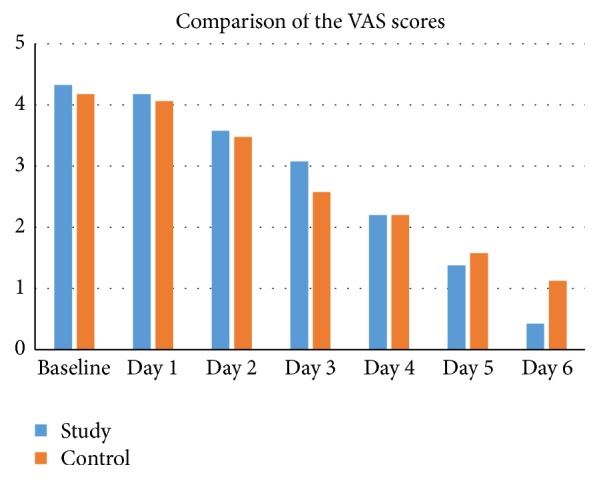
VAS scores of the PKT and control groups measured each day.

**Figure 2:**
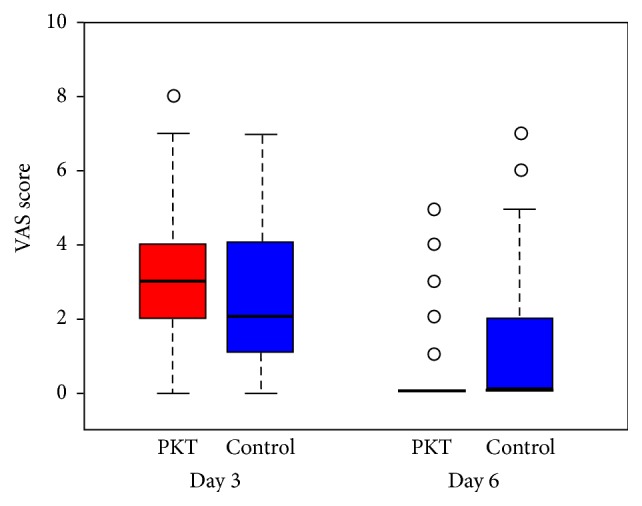
VAS scores of the PKT and control groups at two follow-ups.

VAS scores of the PKT and control groups were compared using the Wilcoxon test. Only the difference at day 6 was found to be significant (Wilcoxon test, *p* < 0.05); see Table 2 and Figures 1 and 2.

#### 3.2.4. Area of Ulcer

**Figure 3:**
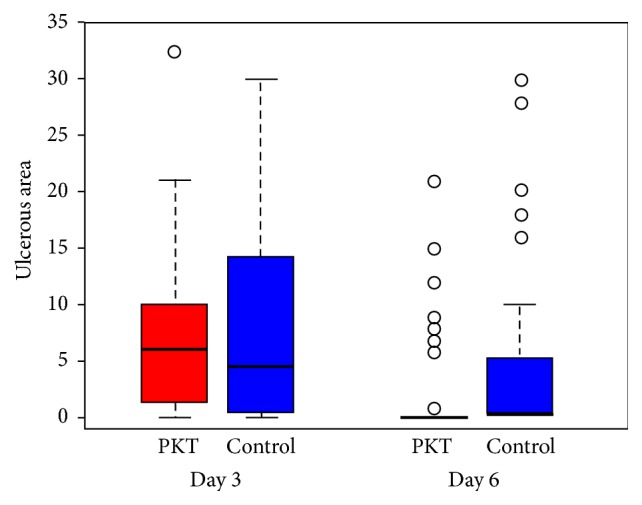
Target ulcer areas of the PKT and control groups at two follow-ups.

The difference in the area of the ulcer between the two groups was not significant at day 3. However, the PKT group had significantly smaller ulcers at day 6; see Table 3 and Figure 3.

#### 3.2.5. Degree of Exudation and Hyperemia

Differences in the degree of exudation and hyperemia between the PKT group and test group were found to be significant at day 6; see Table 4.

#### 3.2.6. Analysis of Possible Confounding Factors

During the study period, there were no medical conditions or use of drugs reported by either group. The degree of compliance was similar for the two groups (*p* > 0.05). See Table 5.

#### 3.2.7. Adverse Events

An adverse event was reported in the PKT group. The participant developed a round-shaped exfoliation lesion of the lower lip mucosa with a diameter of 1.5 cm. The lesions possible relationship to the experimental toothpaste was considered and it was suggested that the participant reduces the amount of toothpaste used for teeth brushing. The lesion vanished 6 days later. Eight months after the clinical trial, no other adverse events had been reported to the investigators. The adverse event rate of 1.25% was not significant.

## 4. Discussion

RAUs are the most common oral mucosal disease and usually manifest as pain or discomfort of the oral mucosa that is not accompanied by systemic symptoms. The etiology of RAUs is still unclear. However, many studies suggest that RAUs can be caused by a number of factors [33], such as endocrinal or immunological abnormality, trauma, smoking, stress, use of nonsteroidal anti-inflammatory drugs, nutritional deficiency of vitamin B, or hereditary tendencies [34].

In recent years, pharmaceutical companies have started to investigate the possible efficacy of drugs that contain natural herbal extracts to treat RAUs [35]. Herbal medicine has been reported to promote the healing of RAUs, as well as reduce the healing period [36]. The use of toothpaste containing herbal medicines has been gradually accepted as there is evidence that it has a safety advantage over purely chemical toothpastes [37].

The ingredients of Pudilan have been shown to be safe [38, 39] and have various pharmacological activities. Dandelion extract can affect TNF-*α* and IL-6 expression and has been shown to reduce inflammation through inhibiting the production of IL-1 [40, 41].* Radix isatidis* has been shown to inhibit the endotoxin-induced secretion of NO, PGE-2, and IL-6 by macrophages, thereby preventing the spread of inflammation [42]. The herb* Bunge corydalis* has been shown to promote injury recovery by improving local blood circulation, inhibiting inflammation, reducing edema, and promoting the formation of granulation tissue. It has also been shown to inhibit the expression of TNF and IL-6, thus enhancing recovery from infection [43].* Scutellaria baicalensis* was reported to be able to remove free radicals, have antioxidant and anti-inflammatory effects, inhibit replication of* Staphylococcus aureus* and* Streptococcus hemolyticus*, and inhibit the migration of inflammatory cells, thereby alleviating local inflammation [44, 45]. Propolis extract has been shown to inhibit growth of pathogenic microorganisms, inflammatory reactions, and ulcers and to alleviate pain [46]. Gallnut extracts have been shown to promote coagulation and have antimicrobial and antiviral activities [41]. Furthermore, gallnut extracts have also been shown to reduce the expression of proinflammation cytokines such as TNF-*α*, IL-6, and IL-1*β* [47]. One study of rats injected with* Radix isatidis* oral liquid or with 5-fluorouracil reported that the ratio of chemotactic factors in the blood plasma of both groups was higher after 8 weeks [48].

In the present study, the healing rate of the PKT group, which used Pudilan extract toothpaste, was significantly higher than that of the control group, indicating that the Pudilan extract was efficacious in treating MiAUs over a short period. The VAS score of the PKT group at 6 days' follow-up was lower than that of the control group (*p* < 0.05) and, additionally, the change in VAS scores between the beginning and end of the study was higher for the PKT group than for the control group, The PKT group was also found to perform better with respect to control of exudation, hyperemia, and reduction in ulcer area. There was only one minor adverse event reported during the study period, which was not statistically significant, indicating the presumptive safety of the PKT.

Pudilan extract has anti-inflammatory effects that are mediated by inhibition of TNF-*α* and IL secretion and reducing the release of inflammatory mediators such as NO and PGE-2 [49]. Pudilan extract can also inhibit bacterial growth by interfering with ATP synthesis, achieve antipyretic analgesic effects by reducing arachidonic acid production, and alleviate exudation by promoting protein precipitation [44, 47, 50]. This extract has also been reported to have detoxifying and antiedema effects and has been used to treat furunculosis, parotitis, pharyngitis, lymphadenitis, and tonsillitis [51–54].

Toothpaste is a widely used and affordable product for daily oral health care. Its low production cost gives considerable advantages for promoting oral health while reducing expensive dental care. Recently, a number of toothpastes with specific functions have entered the market, and an increasing number of individuals tend to choose a toothpaste that contains herbal medicines [55]. Yunnan Baiyao toothpaste has previously been reported to be safe and to have good short-term treatment efficacy for MiAUs [56]. However, that study did not measure the degree of exudation or hyperemia. The results of the present study suggest that PKT alleviates pain, inhibits inflammation, and promotes ulcer healing, thus improving quality of life for MiAU patients.

Based on the various indices measured in the present study, PKT toothpaste promoted healing of ulcers and reduced pain in MiAU patients. Further investigations including more MiAU patients and an extended observation period are required to better reveal the treatment efficacy and safety of Pudilan extract.

## Figures and Tables

**Table 1:** Superiority test of the healing rate in the two groups.

Group	*N*	Target ulcer healed	Target ulcer not healed	Healing rate (%)	95% CI (HO: T − C ≤ 0)	*p* value
Study	40	32	8	80	[0.125, +∞]	0.002
Control	40	20	20	50		

Control	40	20	20	50	[−0.475, +∞]	0.998
Study	40	32	8	80		

**Table 2:** VAS scores of the PKT and control groups.

Time of measurement	Group	*N*	Mean	Std	95% CI	*p* value
Day 3	Study	40	3.08	1.99	2.46–3.69	0.2147
	Control	40	2.58	2.26	1.87–3.28	

Day 6	Study	40	0.43	1.13	0.075–0.78	0.0156
	Control	40	1.13	1.77	0.576–1.674	

**Table 3:** Comparison of the ulcerous area in the PKT and control groups.

Time of measurement	Group	*N*	Mean	Std	95% CI	Wilcoxon *p* value
Day 3	Study	40	6.68	6.55	4.65–8.71	0.9885
	Control	40	8.67	9.48	5.73–11.60	

Day 6	Study	40	1.98	4.76	0.50–3.45	0.0108
	Control	40	4.91	8.69	2.22–7.61	

**Table 4:** Comparison of the degree of exudation and hyperemia in the PKT and control groups.

	Time of measurement	Score	Number of volunteers (% of the group)		Wilcoxon test *p* value
			Study group	Control group	
Exudation	Day 3	0	3 (7.5%)	9 (22.5%)	0.9471
		1	11 (27.5%)	6 (15.0%)	
		2	21 (52.5%)	15 (37.5%)	
		3	5 (12.5%)	10 (25.0%)	
	Day 6	0	32 (80.0%)	22 (55.0%)	0.0180
		1	3 (7.5%)	7 (17.5%)	
		2	5 (12.5%)	9 (22.5%)	
		3	0 (0%)	2 (5.0%)	

Hyperemia	Day 3	0	6 (15.0%)	8 (20.0%)	0.7803
		1	21 (52.5%)	19 (47.5%)	
		2	13 (32.50%)	13 (32.5%)	
		3	0 (0%)	0 (0%)	
	Day 6	0	33 (82.5%)	22 (55.0%)	0.0100
		1	6 (15.0%)	16 (40.0%)	
		2	0 (0%)	2 (5.0%)	
		3	1 (2.5%)	0 (0%)	

**Table 5:** Comparison of degree of compliance in the two groups.

Group	*N*	Mean	Min	25th percentile	75th percentile	Max	Std	95% CI	Wilcoxon *p* value
Study	40	100.2	91.7	100.0	100.0	116.6	3.510	99.1–101.3	0.1701
Control	40	101.0	83.3	100.0	100.0	116.7	5.056	99.5–102.6	
